# Incidental Detection of Miliary Tuberculosis During Laparoscopic Cholecystectomy: A Case Report

**DOI:** 10.7759/cureus.108596

**Published:** 2026-05-10

**Authors:** Md. Akbar Khan, Saddiqa Rahman, Nasheeta Usman

**Affiliations:** 1 Department of General Surgery, Madhubani Medical College, Madhubani, IND

**Keywords:** granulomatous inflammation, incidental intraoperative finding, laparoscopic cholecystectomy, miliary tuberculosis, peritoneal tuberculosis

## Abstract

Miliary tuberculosis is a rare but potentially severe form of disseminated tuberculosis resulting from the hematogenous spread of *Mycobacterium tuberculosis*, leading to the widespread granulomatous involvement of multiple organ systems. Peritoneal tuberculosis represents an uncommon manifestation of extrapulmonary tuberculosis and often presents with vague and nonspecific clinical features, frequently mimicking intra-abdominal malignancies and posing a significant diagnostic challenge. We report the case of a 24-year-old immunocompetent female patient who presented with symptomatic cholelithiasis and underwent elective laparoscopic cholecystectomy. Intraoperatively, multiple whitish, millet-sized nodules were incidentally identified over the peritoneum, liver surface, and omentum. Histopathological examination confirmed caseating granulomatous inflammation consistent with tuberculosis. The patient was subsequently started on anti-tubercular therapy and showed good clinical improvement on follow-up. This case highlights the importance of intraoperative vigilance, the role of laparoscopy, and the necessity of histopathological confirmation in differentiating tuberculosis from malignancy in endemic regions.

## Introduction

Tuberculosis continues to be a major global health problem, particularly in developing countries such as India, where it remains endemic and contributes significantly to the healthcare burden. Extrapulmonary tuberculosis accounts for approximately 15-20% of all tuberculosis cases, and among these, abdominal tuberculosis constitutes nearly 11-16% of cases [1,2]. Peritoneal tuberculosis is a relatively uncommon form, accounting for a small proportion of abdominal tuberculosis, and may arise through hematogenous dissemination from a primary pulmonary focus, reactivation of latent infection, or contiguous spread from adjacent infected organs [3,4].

Miliary tuberculosis represents a disseminated form of the disease characterized by the widespread hematogenous spread of *Mycobacterium tuberculosis*, resulting in the formation of numerous small granulomas resembling millet seeds in various organs such as the lungs, liver, spleen, and peritoneum [1,3]. The disease spectrum ranges from acute fulminant illness to indolent or even asymptomatic forms, particularly in immunocompetent individuals, making diagnosis challenging. Peritoneal tuberculosis is notorious for its ability to mimic intra-abdominal malignancies such as peritoneal carcinomatosis and ovarian carcinoma, both clinically and radiologically [5,6]. Patients may present with nonspecific symptoms, including abdominal pain, distension, fever, or weight loss, while imaging findings such as ascites, peritoneal thickening, and omental caking lack specificity. As a result, preoperative diagnosis is often difficult and may only be established intraoperatively or through histopathological evaluation [2,6].

Diagnostic laparoscopy has emerged as an important modality in such cases, allowing the direct visualization of peritoneal surfaces and enabling the targeted biopsy of suspicious lesions, thereby significantly improving diagnostic accuracy [7,8]. This case highlights the incidental intraoperative detection of miliary tuberculosis in an immunocompetent patient undergoing laparoscopic cholecystectomy for symptomatic gallstone disease.

## Case presentation

A 24-year-old immunocompetent female patient presented with a history of recurrent right upper quadrant abdominal pain for several months. The pain was intermittent, was colicky in nature, and aggravated after meals, consistent with biliary colic. There was no associated history of fever, weight loss, anorexia, night sweats, or chronic cough. The patient had no prior history of tuberculosis, no known contact with tuberculosis patients, and no significant comorbidities.

On clinical examination, the patient was hemodynamically stable with normal vital parameters. General physical examination did not reveal pallor, icterus, or lymphadenopathy. Abdominal examination revealed a soft, non-distended abdomen with mild tenderness in the right hypochondrium. Murphy's sign was negative, and there was no guarding, rigidity, or palpable mass. Examination of other systems was unremarkable.

Routine laboratory investigations revealed hemoglobin of 11.8 g/dL, a total leukocyte count of 7,600/mm³, a differential leukocyte count showing neutrophils 64%, lymphocytes 28%, eosinophils 3%, and monocytes 5%, and a platelet count of 2.74×10⁵/mm³, all within normal limits. The erythrocyte sedimentation rate (ESR) was mildly elevated at 32 mm in the first hour, suggestive of an underlying chronic inflammatory process. Liver function tests were within normal limits, including total bilirubin 0.8 mg/dL, direct bilirubin 0.2 mg/dL, aspartate aminotransferase (AST) 28 U/L, alanine aminotransferase (ALT) 31 U/L, alkaline phosphatase 118 U/L, total protein 7.1 g/dL, and serum albumin 4 g/dL. Renal function tests and serum electrolytes were also within normal ranges, including blood urea nitrogen (BUN) 18 mg/dL, serum creatinine 0.8 mg/dL, serum sodium 138 mEq/L, serum potassium 4.1 mEq/L, serum chloride 102 mEq/L, and serum bicarbonate 24 mEq/L.

A preoperative chest X-ray was performed and appeared normal, with no evidence of pulmonary infiltrates, cavitary lesions, pleural effusion, hilar lymphadenopathy, or other radiological features suggestive of pulmonary tuberculosis.

**Figure 1:**
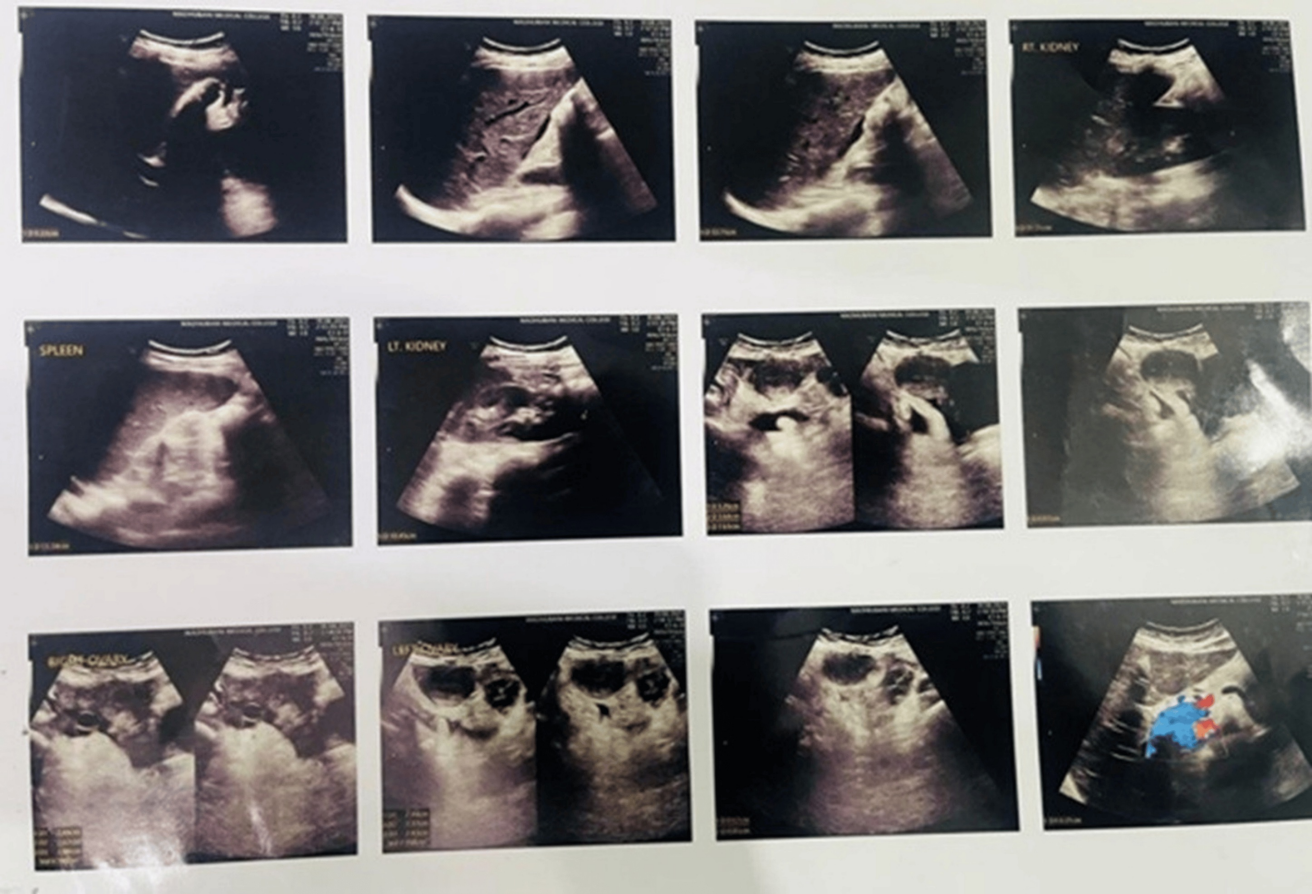
Ultrasonographic images showing gallbladder calculi with posterior acoustic shadowing and mild pericholecystic fluid

Ultrasound of the whole abdomen shows a normal liver, pancreas, kidneys, uterus, and urinary bladder. The gall bladder contains multiple small calculi (largest ~2.8 mm) with mild pericholecystic free fluid but no definite signs of cholecystitis. The spleen is enlarged (splenomegaly). The right ovary appears bulky with multiple developing follicles, while the left ovary is normal. There is mild free fluid in the pouch of Douglas, with no evidence of ascites, masses, or bowel abnormalities. Overall findings are suggestive of cholelithiasis with mild pericholecystic fluid, splenomegaly, and mild free fluid in the pouch of Douglas (Figure 1). No radiological features suggestive of tuberculosis or malignancy were identified preoperatively. Based on these findings, a diagnosis of symptomatic cholelithiasis was made, and the patient was planned for elective laparoscopic cholecystectomy.

**Figure 2:**
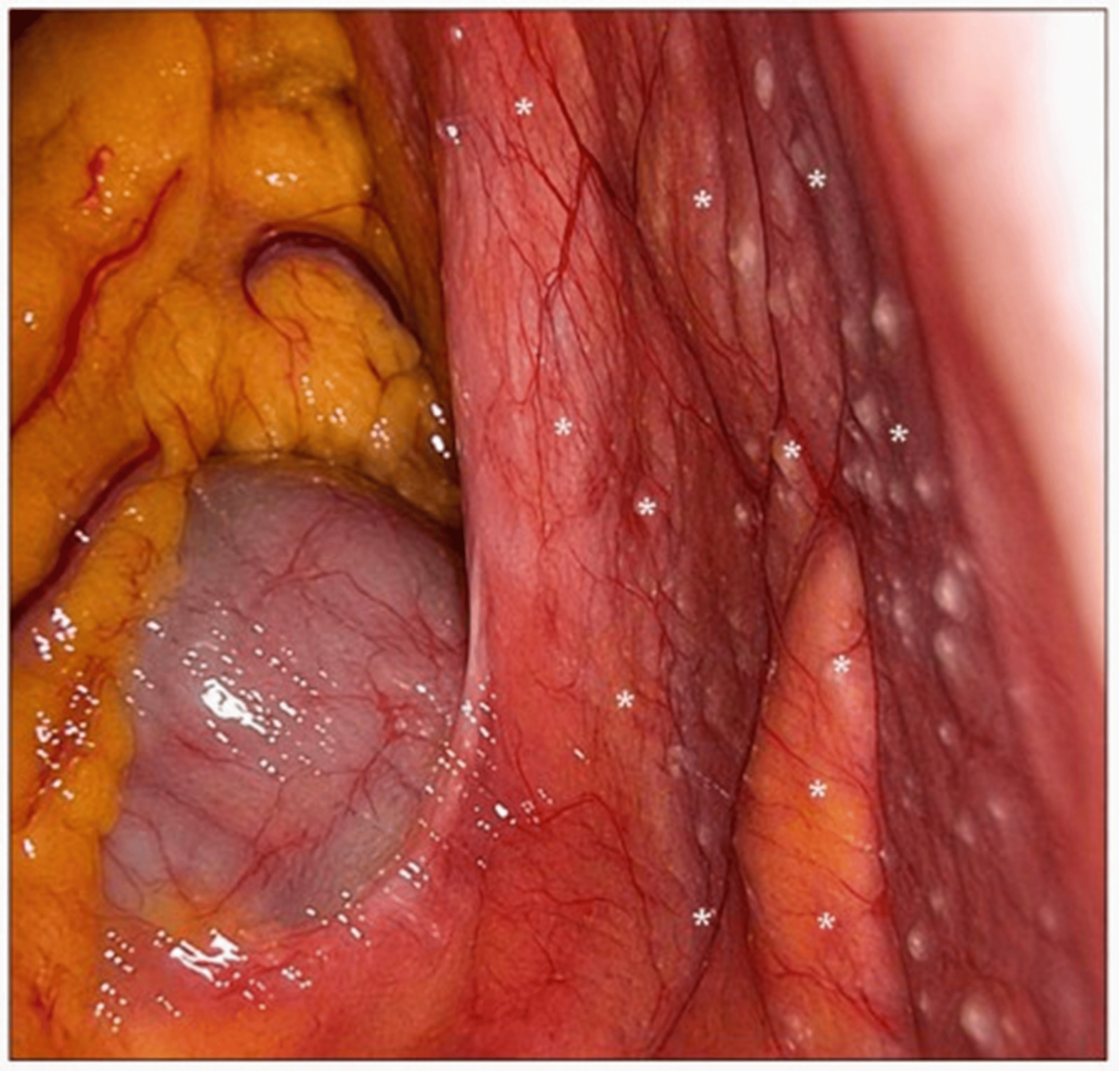
Laparoscopic view showing multiple miliary tubercles (*) over the peritoneum

**Figure 3:**
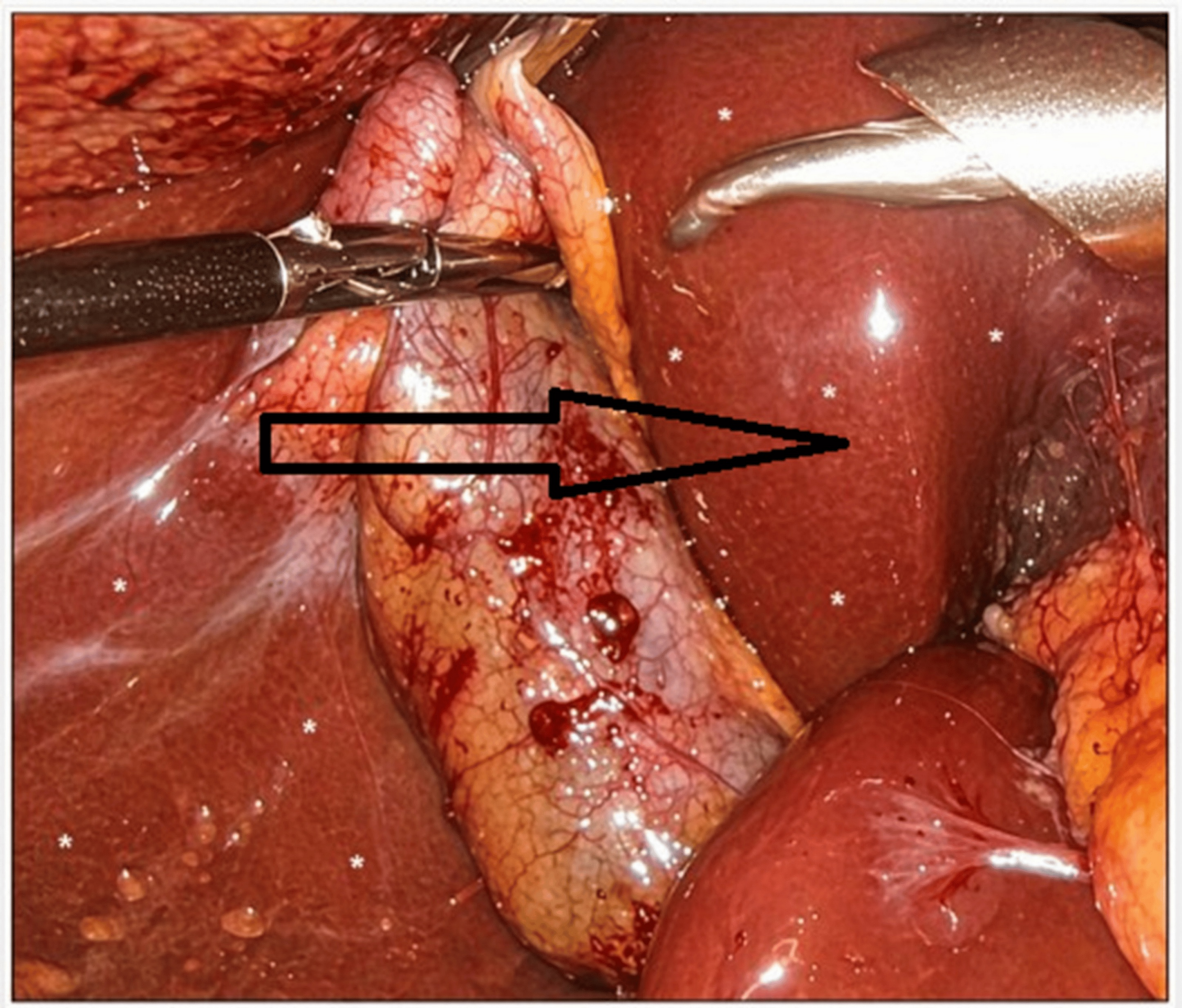
Intraoperative view showing multiple miliary nodular deposits (* and arrow) over the liver surface

**Figure 4:**
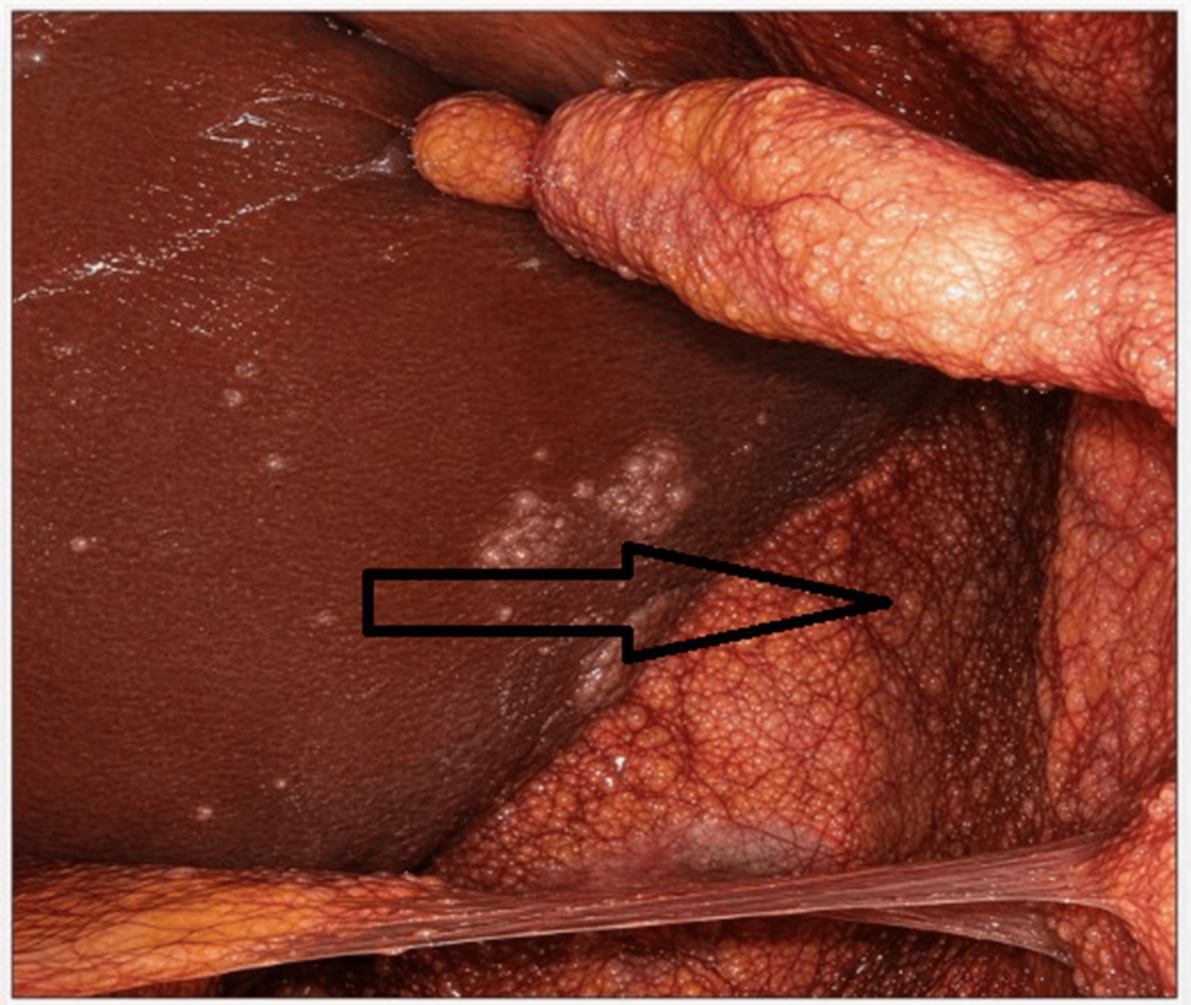
Intraoperative view showing multiple miliary tubercles (arrow) over the omentum

During laparoscopic cholecystectomy, after establishing pneumoperitoneum and gaining access to the peritoneal cavity, multiple whitish, discrete, millet-sized nodules measuring approximately 1-5 mm were observed diffusely distributed over the parietal peritoneum, liver surface, and omentum. These nodules had a characteristic miliary appearance, raising strong intraoperative suspicion of peritoneal tuberculosis, although peritoneal carcinomatosis was also considered as a differential diagnosis. There were no significant ascites, dense adhesions, or obvious mass lesions. The gallbladder appeared consistent with chronic calculous cholecystitis. The intraoperative findings are depicted in Figures 2-4.

**Figure 5:**
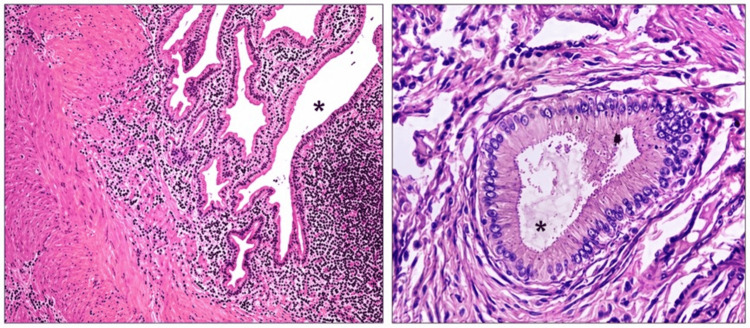
Photomicrographs showing gallbladder wall histology with * marks Left: Low-power view demonstrating mucosal hyperplasia with the formation of Rokitansky-Aschoff sinuses extending into the muscular layer along with dense chronic inflammatory infiltrate. Right: High-power view depicting a cystically dilated sinus lined by columnar epithelium surrounded by fibromuscular stroma, consistent with chronic cholecystitis.

The laparoscopic cholecystectomy was completed uneventfully. Multiple biopsy samples were obtained from the peritoneum, omentum, and liver surface for histopathological evaluation. Histopathological examination of the cholecystectomy specimen (gallbladder) shows multiple blackish calculi with a congested outer surface and greenish velvety mucosa. Microscopy reveals denuded to flattened mucosa with chronic inflammatory infiltrate composed predominantly of lymphocytes and plasma cells, along with Rokitansky-Aschoff sinuses, muscular hypertrophy, and serosal fibrosis with vascular congestion. Multiple confluent epithelioid cell granulomas with multinucleated giant cells are noted in the serosa. No evidence of dysplasia or malignancy is seen. Overall features are suggestive of granulomatous cholecystitis with cholelithiasis, and clinical, radiological, and microbiological correlation (including evaluation for tuberculosis and other granulomatous diseases) is advised. The histopathological features are shown in Figure 5.

The postoperative period was uneventful, and the patient recovered well from surgery. Following the confirmation of the diagnosis, the patient was initiated on standard anti-tubercular therapy consisting of isoniazid, rifampicin, pyrazinamide, and ethambutol for the intensive phase of two months, followed by isoniazid and rifampicin for the continuation phase of four months [1,4]. The patient tolerated the treatment well without any adverse effects. On follow-up, she showed significant clinical improvement with complete resolution of abdominal pain and no new symptoms. At present, the patient is continuing anti-tubercular therapy and remains clinically stable with no complications.

## Discussion

Miliary tuberculosis represents a disseminated form of tuberculosis resulting from the hematogenous spread of *Mycobacterium tuberculosis*, leading to the widespread involvement of multiple organ systems [1,3]. Although it is more commonly associated with immunocompromised states, it can also occur in immunocompetent individuals, as demonstrated in this case. The absence of classical systemic features such as fever, weight loss, and night sweats often contributes to delayed or incidental diagnosis.

Peritoneal tuberculosis is an uncommon manifestation of abdominal tuberculosis and is traditionally classified into wet ascitic, fibrotic-fixed, and dry plastic types [2,4]. The dry plastic type, characterized by multiple peritoneal nodules with minimal or no ascites, correlates with the intraoperative findings observed in this patient. The absence of ascites and nonspecific clinical presentation further complicated the preoperative diagnosis.

A major diagnostic challenge in such cases is differentiating peritoneal tuberculosis from peritoneal carcinomatosis. Both conditions may present with multiple nodular deposits over the peritoneum and omentum and may appear identical intraoperatively. Several studies have documented that peritoneal tuberculosis can closely mimic malignancy, leading to potential misdiagnosis and unnecessary extensive surgical interventions if biopsy is not performed [5-7].

Radiological imaging modalities such as ultrasound and computed tomography are often unable to reliably distinguish between these conditions due to overlapping features such as peritoneal thickening, ascites, and omental involvement. In this case, preoperative imaging did not reveal any findings suggestive of tuberculosis, highlighting the limitations of radiological investigations [2,6].

Diagnostic laparoscopy plays a crucial role in such scenarios by allowing the direct visualization of peritoneal lesions and facilitating targeted biopsy. The typical laparoscopic findings in peritoneal tuberculosis include multiple whitish nodules, peritoneal thickening, and adhesions [7,8]. Histopathological examination remains the gold standard for diagnosis, with the presence of caseating granulomas and Langhans giant cells being characteristic findings [2,6].

Early diagnosis and prompt initiation of anti-tubercular therapy are essential to prevent complications such as adhesions, intestinal obstruction, perforation, and systemic dissemination. Most patients respond well to therapy when diagnosed early, as observed in this case. This case underscores the importance of maintaining a high index of suspicion in endemic regions and performing a biopsy of suspicious intraoperative findings to ensure accurate diagnosis and appropriate management.

## Conclusions

This case highlights the incidental detection of miliary tuberculosis during laparoscopic cholecystectomy in an immunocompetent patient with no classical clinical features of tuberculosis. It emphasizes the importance of intraoperative vigilance and routine biopsy of suspicious lesions. Histopathological confirmation remains essential for diagnosis, and early initiation of anti-tubercular therapy leads to favorable clinical outcomes.
